# Nutrition’s Role in Quality Healthcare in the United States: Opportunities and Education for Pharmacists to Take a Bite of the Apple and Strengthen Their Skills

**DOI:** 10.3390/pharmacy12040103

**Published:** 2024-06-29

**Authors:** Jeff Cai, Andie Lee Gonzalez, Mary Beth Arensberg

**Affiliations:** 1 College of Pharmacy, The University of Texas, Austin, TX 78712, USA; 2 Abbott Nutrition Division of Abbott, Columbus, OH 43219, USA

**Keywords:** pharmacist, diet and nutrition, food is medicine, nutrition education, pharmacy continuing education, health policy

## Abstract

With global chronic disease rates on the rise, diet and nutrition remain pivotal yet under-appreciated aspects of healthcare, including in pharmacy practice. This perspective paper delves into how current United States health policies support nutrition’s role in healthcare and its integration into pharmacy practice. The paper also reviews the landscape of nutrition education and training for pharmacists, pharmacy roles in multidisciplinary teams and interprofessional nutrition care, and the opportunities for post-graduate nutrition-focused certification, training, and continuing education. It advocates for a paradigm shift towards greater emphasis on nutrition within pharmacy practice, to improve skills and benefit quality patient nutrition care.

## 1. Introduction

Against a backdrop of ever-increasing rates of chronic disease worldwide, the focus is typically on medical and pharmaceutical interventions. Diet and nutrition are often overlooked, yet they should be considered—including by pharmacists. Diet is one of the top three factors contributing to the global disease burden [1]; in the United States (U.S.), poor health from poor nutrition is a leading cause of both adult morbidity and mortality [2]. Nutrition also plays a pivotal role in pediatric health, serving as a foundation for proper growth and development and influencing long-term health outcomes in areas like immune function and chronic disease prevention [3]. Such realities underscore the importance of nutrition as an area of interprofessional development for pharmacy education and practice. Pharmacists can be instrumental in providing guidance on nutrition supplements and dietary needs and collaborating with other healthcare professionals to ensure a holistic approach to nutrition [4]. This perspective paper identifies how today’s U.S. health policy environment supports nutrition’s important role in equitable, quality U.S. healthcare and outlines potential intersections with the scope of pharmacy practice. Further, we describe the current state of nutrition education and training for pharmacists in the U.S. and pharmacy roles in nutrition. Finally, this perspective paper explores opportunities for post-graduate nutrition-related certifications, training, and continuing education, positioning pharmacists to “take a bite of the apple” and strengthen their nutrition skills to better support patient health outcomes and care.

## 2. U.S. Policy Developments Advancing Nutrition’s Role in Quality Healthcare

Nutrition is vital for sustaining life. It also has a fundamental role in helping prevent and treat chronic disease. Nearly half of all American adults have at least one chronic disease that may be preventable, like type 2 diabetes, high blood pressure, cardiovascular disease, and cancer [5]. Many of these chronic diseases are related to an inadequate-quality diet and physical inactivity [6]. An additional factor linked to chronic disease is food insecurity, defined as a “household-level economic and social condition of limited or uncertain access to adequate food” [7]. The U.S. rate of food insecurity increased to 12.8% in 2022 [8]. Obesity is another chronic disease associated with diet and food insecurity. By 2030, almost one in two U.S. adults will have obesity, with about one in four having severe obesity. Further, in an estimated 29 states, obesity prevalence in adults will be over 50% by 2030 [9]. Obesity is one of the most common pediatric chronic diseases, affecting more than 14.4 million American children and adolescents [10].

These startling statistics are driving U.S. policy actions specific to nutrition and food insecurity. In 2022, the first White House nutrition conference in over 50 years was held. Following this White House Conference on Hunger, Nutrition and Health, the Biden Administration called for a “whole-of-government” and “whole-of-America” approach to addressing challenges related to hunger, nutrition, and health. One particular recommended action was that “Health professional schools (e.g., medical, dental, pharmacy, nursing, social work, public health, physician’s assistants, physiology, exercise science, etc.) and licensing boards should expand nutrition education in graduate medical education curriculums, board exams, and post-graduate training” [11]. 

The Biden Administration has already taken several steps to integrate screening for poor nutrition or malnutrition and food insecurity into quality healthcare. Specifically, the U.S. Centers for Medicare & Medicaid Services (CMS) adopted the first electronic, clinical nutrition-focused quality measure in any CMS payment program in 2022 [12]. This quality measure, the Global Malnutrition Composite Score (GMCS), was approved as a measure of health equity and incentivizes hospitals to identify malnutrition, including malnutrition resulting from food insecurity [13]. CMS also included nutrition and food insecurity risk screening as a Merit-based Incentive Payment System (MIPS) improvement activity under the 2022 Physician Fee Schedule Final Rule [14] and then later as an improvement activity for several MIPS value pathways (MVPs) [15]. 

Such U.S. federal government policy efforts provide the frameworks for nutrition-focused quality improvement programs (QIPs) that document how nutrition screening and interventions can help improve health outcomes and reduce healthcare costs. For example, Hong et al. [16] investigated a nutrition-focused QIP incorporating oral nutrition supplements (ONSs) that were condition-specific and used as part of daily dietary intake in the outpatient setting. The results showed an 11.6% decrease in patients who used healthcare resources during the 90-day time of the study, leading to net savings of nearly USD 500 per patient. In the hospital setting, Siegel et al. [17] showed a nutrition-focused QIP with ONS decreased by 0.88 days the length of stay for patients who were at nutrition risk, as compared to patients who were not at nutrition risk. Further, the Malnutrition Quality Improvement Initiative (MQii), formed over a decade ago to increase high-quality, evidence-based, and patient-driven care, provides nutrition-focused resources for quality improvement, including an interprofessional toolkit to help better identify and manage malnourished, adult, hospital patients [18].

Food is medicine initiatives are another growing U.S. federal government policy area advancing nutrition’s role in quality healthcare. The U.S. Department of Health and Human Services held its inaugural Food is Medicine Summit in 2024 [19]. This summit was part of the Department’s larger initiative to unify and advance collective action on incorporating consistent access to nutrition and diet-related resources across communities and systems [5]. CMS has also started testing several food is medicine initiatives in its Medicare and Medicaid programs [20,21]. 

In addition, there is recent evidence of pharmacists’ interest in nutrition and their role in helping address nutrition in chronic disease. In the article “The Pharmacist of the Future”, Van Antwerp et al. [22] identified that food is medicine developments could, in part, help pharmacists to intervene upstream through coaching on lifestyle and diet. Similarly, during a session at the American Pharmacists Association (APhA) 2023 Annual Meeting & Exposition, the grocery store was identified as a unique health destination that could offer an opportunity for collaboration and interprofessional care between pharmacists and in-store dietitians [23]. The session reported results of a randomized controlled trial evaluating an individualized supermarket and web-based intervention targeting nutrition (SuperWIN). The SuperWIN study showed in-aisle education significantly increased adherence to a hypertension diet, compared to traditional nutrition counseling alone [24].

Others have described the development of produce prescriptions and food pharmacies as innovations that may be linked to state-licensed pharmacies [25] and are thinking through the opportunities for pharmacists in such programs [26,27]. Further, the American Society of Health-System Pharmacists (ASHP) has included nutrition as part of both the upstream and downstream prevention-related factors discussed in their current Statement on the Pharmacist’s Role in Public Health [28]. More recently, the National Association of Chain Drug Stores, in response to the 2022 White House Conference on Hunger, Nutrition and Health, launched #NourishMyHealth^SM^, started a nationwide public education campaign promoting the connection between nutritious foods and chronic disease [29]. Building on such efforts, potential community pharmacy roles related to food is medicine are described in Table 1.

There has also been exploration of consumer views specific to pharmacists and nutrition. A nationwide poll of over 10,000 U.S. adults found that more than three-fourths (76%) believed pharmacists have a role in “helping patients understand their nutritional choices“ [36]. Pharmacy nutrition education may help strengthen this role. In a separate exploration of consumers’ opinions about a pharmacist’s role in nutrition counseling, it was identified that consumers do not interact with pharmacists as much as they do with other health professionals on nutrition issues. In part, this was because one-third of the consumers surveyed believed pharmacists lacked proper training to consult on nutrition [37].

## 3. Nutrition in Pharmacy Education and Training

To inform our perspective on the current state of nutrition education and training for U.S. pharmacists, we surveyed the medical literature over the last two decades to identify reported nutrition-specific pharmacy education requirements. The U.S. has over 140 colleges and schools of pharmacy with accredited professional degree programs [38]. Currently, there is no defined federal standard requiring nutrition as a core course in U.S. pharmacy school curricula. However, the American College of Clinical Pharmacy (ACCP) regularly publishes a Pharmacotherapy Didactic Curriculum Toolkit “used by colleges and schools of pharmacy as a guide for curricular development.” The most recent 2023 update includes nutrient deficiency/excess and overweight/obesity as tier-one recommendations (students receive sufficient knowledge and skills to enable them to be “practice-ready”). Also included are malabsorptive syndrome and malnutrition prevention/treatment as tier-two recommendations (students receive foundational knowledge and skills but require additional training to be “practice-ready”) [39].

Lim et al. [40] documented 66% of surveyed U.S. pharmacy schools offered at least one course that had nutrition as its primary objective and was related to counseling, dieting, nutrients, diseases, therapies, and/or interactions. They commented this was an increase compared to a 2007 study documenting that just 14% of U.S. pharmacy schools offered coursework on lifestyle strategies promoting public health and disease prevention [41]. Yet, Lim et al. [40] reported that survey respondents’ overall perception was that pharmacy student nutrition training is relatively inadequate. This perception is in line with the results from a survey of U.S. pharmacy students, where just 13.7% reported they had received education in nutrition, even though 82.9% of those surveyed believed that nutrition should be a part of the degree curriculum for pharmacy [42].

Thus, nutrition education in U.S. pharmacy schools appears to be limited. There may be some focus on counseling, diseases, and therapies, but education at the practice-ready skill level is likely specific to certain conditions, such as nutrient deficiencies/excesses and overweight/obesity, as identified in the 2023 ACCP Pharmacotherapy Didactic Curriculum Toolkit [39]. The culinary medicine approach, which is gaining traction in medical school [43] and interprofessional [44] curricula, provides a potential framework for strengthening and broadening pharmacy student nutrition education [45]. Such courses are experiential-based and offer hands-on culinary skill development along with strategies for translating nutrition science into lifestyle practices that help prevent disease and improve health outcomes [43]. Culinary medicine curricula have been shown to improve medical student knowledge and self-efficacy in nutrition counseling [43,46]. In a different model of increasing nutrition training in pharmacy education, at least one U.S. university offers a combined Doctor of Pharmacy (PharmD)/Master of Science in Nutrition program [47]. 

In other developed countries such as Australia, pharmacists are seen as “first and foremost medication specialists and suppliers, ideally positioned within the healthcare setting to encourage and support positive lifestyle choices in the community” [48]. Although considered among the most accessible healthcare professionals, pharmacists themselves have recognized their lack of nutrition knowledge. Specifically, in the same Australian survey, pharmacists were shown to have a significant variation in nutrition education, mentioning their training was inconsistent and usually not something taught in their training. The overwhelming majority of pharmacists (95.7%) agreed “they are well-placed to assist in disease burden reduction through nutrition education” while, at the same time, nearly all (98.4%) believed “their knowledge needed improvement” [48]. Additional education was viewed as a way to enhance nutrition knowledge without significant time requirements and as a way to improve a pharmacist’s counseling practices [48]. Similarly, in a survey of pharmacists in Ireland, over three-fourths (78.1%) of respondents agreed they “would like further nutrition education to support themselves in their roles as pharmacists” [49].

Licensing requirements can influence pharmacy education too. In reviewing U.S. state pharmacy licensure, we found no documentation of any U.S. state licensure requirements specifically related to nutrition and disease. Rather, nutrition is often mentioned in more general terms. For example, per the Texas State Board of Pharmacy (TSBP), nutrition is grouped together with nonprescription products, devices, dietary supplements, and traditional nondrug therapies. The knowledge requirements for Texas pharmacy licensure are that the pharmacist or pharmacy intern is able to communicate and demonstrate competence in counseling patients for a condition, intended use, adverse effects, and triaging the need for treatment/referral related to the use of these products [50,51]. It is important to note that some states have specific scopes of practice laws that require a nutrition/dietetics license to provide nutrition counseling and medical nutrition therapy (nutrition-based treatment provided by a registered dietitian [52]). 

Nutrition support is one area where pharmacists have traditionally held essential roles and the Texas pharmacy licensure requirements specifically include mention of compounding nutrition solutions. However, there has also been a call for enhanced pharmacy education in nutrition support. Salman et al. described deficiencies in both pharmacists’ nutrition support knowledge as well as skills, identifying, for example, that nutrition support rotations are not a required part of the post-graduate year-one (PGY1) residencies accredited by ASHP [53].

## 4. Pharmacists as Part of Multidisciplinary Teams, Patient-Based Care, and Interprofessional Education Related to Nutrition

Nutrition support teams have typically included pharmacists, physicians, dietitians, and nurses with clinical nutrition specializations and are an example of multidisciplinary teams that allow for each professional to leverage their own individual expertise [54]. As members of the nutrition support team, pharmacists develop and implement parenteral nutrition protocols as well as provide recommendations on parenteral nutrition composition, parenteral admixture stability and compatibility, and enteral and parenteral nutrition drug and medication interactions. They may also oversee the logistics for parenteral nutrition and support ongoing research on complex nutrition therapy [54].

Pharmacists also play a key role in patient-based care, enhancing the delivery of care and patient experiences [55,56]. The World Health Organization has emphasized how healthcare professionals with different expertise and training—including pharmacists—can help support comprehensive services and the highest quality of care across healthcare settings as they work together with patients, their families, and communities [57]. Collectively, through interprofessional education, pharmacists along with other healthcare professionals can learn the skills needed to positively affect patient care and reinforce that nutrition is important for overall health [4,58]. At the curricular level, there is evidence that interprofessional education on nutrition and lifestyle coaching can improve student confidence (including for pharmacy students) in the coaching of families [59]. Clearly, pharmacists have a key responsibility alongside other healthcare providers in meeting patient primary care needs [60]. However, there is limited information in general about pharmacists’ views on interprofessional education or collaborative practice [55].

## 5. Pharmacy Opportunities for Nutrition-Related Certification and Training

Although there is limited nutrition education at the pharmacy school curriculum level, post-graduate pharmacists can pursue various certification and training programs (Table 2) and continuing education courses to enhance their nutrition understanding and expertise and, thus, better meet patient nutrition needs. For example, in nutrition support, an education-based license is available with the U.S. Board Certified Nutrition Support Pharmacy (BCNSP) Specialty Certification. This certification, obtained through and sponsored by the Board of Pharmacy Specialties [61], is for those who may be pursuing a specialty that works closely with various types of nutrition support such as enteral or total parenteral nutrition (TPN). Certification eligibility requires at least three years of nutrition support pharmacy practice or completion of a post-graduate year-two (PGY2) pharmacy residency in nutrition support. However, unfortunately, in the U.S., PGY2 nutrition support training positions are not available any longer [53].

Another nutrition-support-focused pathway is available through the National Board of Nutrition Support Certification (NBNSC), which is an independent credentialing board that was established by the American Society for Parenteral and Enteral Nutrition (ASPEN) [72]. This credential is the certified nutrition support clinician (CNSC) for licensed pharmacists with at least two years of nutrition support practice experience [63]. It helps validate the pharmacist’s advanced knowledge and experience in promoting optimal nutrition status through direct patient care. ASHP offers a nutrition support certificate that was also developed with ASPEN. The certificate is achieved through completion of a series of online modules but does not have specific requirements for nutrition support practice [64]. Additional education opportunities for nutrition support experience include traineeships and fellowships, like the Emory University Hospital Nutrition Support Pharmacy Fellowship [73], although the prevalence of such programs in the U.S. has declined [53].

Other pharmacy certification programs are available specific to nutrition and chronic disease and lifestyle medicine. These include the Certified Nutrition Specialist (CNS) credential, offered with the American Nutrition Association (ANA) [74]. This certification focuses on personalized nutrition therapy and science-based nutrition practices. Eligibility includes meeting the accredited program and degree requirements and completing 1000 h of Supervised Practice Experience (SPE) with a Board for Certification of Nutrition Specialists-approved supervisor [65]. ASHP offers online certificates in weight management and in diabetes management, which, similar to the ASHP nutrition support certificate, do not have practice-specific requirements [66,67]. The American College of Lifestyle Medicine and American College of Culinary Medicine each have certificates that include both online coursework and hands-on experience [68,69]. Earlier in this paper, it was identified that culinary medicine curricula could also provide a potential framework for strengthening pharmacy student education in nutrition [45]. The U.S. National Institutes of Health Office of Dietary Supplements offers a multi-day research practicum focused on dietary supplements with agency experts as faculty [70]. All of these programs bolster the pharmacist’s expertise in nutrition, contribute to improved patient outcomes through integrated care approaches, and play crucial roles in interprofessional healthcare, helping ensure patients receive optimal nutrition care. Importantly, the nutrition specialist certification programs require recertification after a period of time, often including documented continuing education. 

## 6. Existing and Emerging Platforms for Pharmacy Continuing Education in Nutrition

Pharmacy continuing education (PCE) has evolved to include various platforms that offer accessible, interactive, and up-to-date learning opportunities for pharmacists, including in nutrition. The Accreditation Council for Pharmacy Education (ACPE) sets the standards in pharmacy education through various programs [75]. ACPE is the governing arm for PCE, to ensure that the education available to pharmacists adheres to their guidelines for continuing education.

Looking further into nutrition-specific continuing education and courses available for pharmacists, various programs are offered through multiple organizations and universities. Nutrition-related topics typically range from vitamin supplementation to fluid balancing, toxicities, deficiencies, ONS, enteral and parenteral nutrition products, nutrition access, and nutrition assessment. Although the topics vary in difficulty and content, they are typically focused on pharmacists who practice or plan to practice in hospital or infusion pharmacy-based practices.

The National Association of Boards of Pharmacy (NABP) provides PCE credits through knowledge-based activities that are designed to enhance the professional knowledge and skills of pharmacists [76]. These activities are available as both live and home-study programs, covering a variety of topics. The courses are available in multiple formats, like articles, webinars, and podcasts, catering to different learning preferences and needs. The ASHP is another major platform for pharmacy continuing education [77]. It offers resources for professional development across multiple disciplines within pharmacy. ASHP’s platform also includes live webinars, online courses, and other educational programs, making it a comprehensive resource for pharmacists seeking to advance their careers and stay current in practice. Further, ACPE is a founding member of the Joint Accreditation organization for interprofessional continuing education, meaning that pharmacists can receive credit for interprofessional continuing education (IPCE) that may be available from a number of education providers [78]. 

These different platforms exemplify the shift towards a more flexible learning environment for pharmacy education, enabling pharmacists to maintain and enhance their competencies in a rapidly evolving healthcare landscape [79,80,81]. The platforms also highlight the importance of lifelong learning to ensure the provision of high-quality care and staying updated on the latest advancements in pharmacy practice, including in areas related to nutrition. However, nutrition may not be a common education topic on all these platforms, underscoring the opportunity for the further development of nutrition-focused continuing education for pharmacists.

## 7. Conclusions

Nutrition-focused board certification and continuing education programs are available in the U.S., yet there are no defined requirements for either pharmacy students or pharmacists to include nutrition in their core curriculum or post-graduate education and training. This needs to change to promote optimal nutrition care and address the rising prevalence of diet-related chronic disease.

Pharmacists are vital for inpatient pharmacotherapy management, which can be influenced by a range of factors, from patient vitals to electrolyte levels and food intake. In addition, clinical pharmacists have a critical responsibility in enteral and TPN provision, often through interprofessional collaboration with other healthcare providers, including dietitians, physicians, and nurses. At the community level, pharmacists are trusted professionals who can influence health outcomes by strengthening their expertise and potentially expand their scope of practice in nutrition and lifestyle coaching and interventions. It is time pharmacists take a bite of the apple and leverage nutrition-focused training opportunities and education to improve their skills and benefit quality patient nutrition care.

## Figures and Tables

**Table 1:** Potential community pharmacy roles related to food is medicine.

Role	Description	Barriers	Opportunities
Hosting or partnering with a food pharmacy [30]	Food pharmacies often co-located with other health-related resources Offerings can include: ○ Groceries, ○ Education (including cooking demonstrations and skill development, taste tests, recipe distribution), ○ Cooking equipment, ○ Connecting to other food resources including the U.S. federally-funded Supplemental Nutrition Assistance Program (SNAP) and the Special Supplemental Nutrition Program for Women, Infants, and Children (WIC)	Funding Lack of trust between patients and clinic providers	Offering food can be an incentive to help bring individuals with uncontrolled chronic conditions into a clinic to receive education on medications
Facilitating food is medicine prescriptions [26]	“Prescriptions” for specific foods combined with prescriptions for medications to improve outcomes for individuals with diet-related disease(s) and/or food insecurity	Lack of integrated technology systems, data, and reimbursement to bring food prescriptions to scale	Serve as community point of care to identify when food prescriptions are needed by individuals who have medication prescriptions for chronic diseases Help design technology to manage food prescriptions and collect data Advocate for potential reimbursement mechanisms
Increasing patient awareness about food is medicine-related benefits	Some medical plans provide coverage for certain food is medicine-related benefits	Lack of patient awareness about food is medicine-related benefits	Educate patients about potential: ○ Medicare Advantage and Medicaid programs offering food is medicine benefits including home delivered meals [31,32] and over-the counter benefit card coverage of oral nutrition supplements [33] ○ Employer Health Savings Account (HSA) and Flexible Spending Account (FSA) coverage of oral nutrition supplements [34]
Serving as a community resource	Community pharmacies can be a resource for patients interested in learning more about nutrition and wellness	Space for classes	Work with a local registered dietitian nutritionist to host nutrition classes at the pharmacy Host an event for national nutrition month (March) and spread awareness on social media Recommend vitamin/mineral supplements or oral nutrition supplements to patients at risk of drug-induced nutrient depletion and other nutrition-related effects, including for patients using anti-obesity medications [35]

**Table 2:** Examples of nutrition-related certifications and training programs for pharmacists in the United States (U.S.).

Nutrition-Related Certification or Training	Supporting Organization(s)	Description and Requirements
Board Certified Nutrition Support Pharmacy (BCNSP) Specialty Certification [62]	Board of Pharmacy Specialties	Exam-based certificate for licensed pharmacists with at least 3 years nutrition support pharmacy practice or completion of post-graduate year two (PGY2) pharmacy residency in nutrition support pharmacy Recertification every 7 years
Certified Nutrition Support Clinician (CNSC) [63]	National Board of Nutrition Support Certification	Exam-based certificate offered to licensed pharmacists with at least 2 years of experience in nutrition support practice following professional licensure Recertification every 5 years
Nutrition Support Certificate Program [64]	American Society of Health-System Pharmacists (ASHP) Developed in collaboration with the American Society for Parenteral and Enteral Nutrition (ASPEN)	12 online modules (20 h of continuing professional education (CPE) Passing certification exam
Certified Nutrition Specialist (CNS) [65]	Board for Certification of Nutrition Specialists	PharmD 36 semester credit hours of relevant coursework from U.S. accredited institution Documented achievement of defined practice competencies in 1000 h of supervised practice experience Passing certification exam Recertification every 5 years
Weight Management Certificate [66]	ASHP	20.5 h of on-line Accreditation Council for Pharmacy Education (ACPE) continuing education (CE) incorporating recorded presentations and skill-focused activities Passing certification exam
Diabetes Management Certificate [67]	ASHP	36 h of on-line ACPE CE including diagnosis, classification, pathophysiology of diabetes; facilitating behavior change; use of devices for treatment and monitoring; practical considerations for optimizing glycemic management in inpatient/ambulatory settings Passing certification exam
Lifestyle Medicine Certification [68]	American College of Lifestyle Medicine	PharmD 30 h of online/non-live continuing medical education (CME), 10 h of in-person CME on lifestyle medicine Passing certification exam Recertification every 10 years or ongoing certification
Certified Culinary Medicine Specialist (CCMS) [69]	American College of Culinary Medicine	Licensed practicing pharmacist 60 credit program combination of online coursework and hands-on teaching kitchen modules Completing capstone project Passing certification exam Recertification every 5 years
Mary Frances Picciano Dietary Supplement Research Practicum [70]	National Institutes of Health (NIH) Office of Dietary Supplements	Multi-day educational practicum providing fundamental knowledge on dietary supplements Faculty include U.S. National Institutes of Health (NIH) and Food and Drug Administration (FDA) experts
Nutrition Support Pharmacy Traineeships and Fellowships [71]	Various	Advanced nutrition training through academic clinical institutions, may include research components
